# Supplementary material for: Systemic Uremic Toxin Burden in Autism Spectrum Disorder: A Stratified Urinary Metabolite Analysis
Source: Int J Mol Sci. 2025 Jul 23;26(15):7070. doi: 10.3390/ijms26157070 (PMC12345674; doi:10.3390/ijms26157070)
Supplement: Supplementary file 1 [file ijms-26-07070-s001.zip › ijms-3738355-supplementary.pdf]

Supplementary Materials – Uremic Toxin Study

Table S1. Uremic toxin concentrations in ASD and control children aged 2–5.9 years.

2 – 5.9 yeras	**Control** 2 – 5.9 yeras **(N=14)**	**ASD-**2 – 5.9 yeras **(N=35)**	**ASD/Control ratio**	**p value**
**ADMA**				0.67
Mean (SD)	18.35 (9.16)	20.29 (8.82)	1.11	
Median (Q1, Q3)	16.96 (14.23, 22.50)	18.19 (13.58, 25.74)	1.07	
Min - Max	5.00 - 33.16	5.00 - 39.17		
**SDMA**				0.97
Mean (SD)	42.06 (18.35)	42.43 (19.14)	1.01	
Median (Q1, Q3)	37.93 (28.45, 49.83)	38.93 (26.14, 56.76)	1.03	
Min - Max	20.98 - 81.44	11.37 - 88.90		
**TMAO**				0.06
Mean (SD)	4.59 (3.52)	2.72 (2.85)	0.59	
Median (Q1, Q3)	4.36 (1.56, 7.77)	2.48 (0.05, 3.75)	0.54	
Min - Max	0.05 - 10.38	0.01 - 10.76		
**IS**				0.37
Mean (SD)	79.34 (45.90)	65.35 (38.61)	0.82	
Median (Q1, Q3)	79.11 (42.48, 108.45)	55.09 (36.76, 95.65)	0.70	
Min - Max	10.17 - 155.89	8.26 - 166.23		
**PCS**				0.66
Mean (SD)	58.07 (42.24)	54.52 (46.80)	0.94	
Median (Q1, Q3)	43.72 (26.42, 86.75)	40.42 (18.98, 69.52)	0.92	
Min - Max	7.80 - 143.80	2.35 - 174.60		

Median (Q1, Q3), mean ± SD, range, ASD/Control ratios, and p-values for ADMA, SDMA, TMAO, IS, and PCS in the younger age group. Trends suggest lower TMAO and higher PCS in ASD.

Table S2. Uremic toxin concentrations in ASD and control children aged 6–17 years.

6 – 17 years	**Control** 6 – 17 years **(N=57)**	**ASD-**6 – 17 years **(N=126)**	**ASD/Control ratio**	**p value**
**ADMA**				0.22
Mean (SD)	13.28 (6.17)	14.61 (6.45)	1.10	
Median (Q1, Q3)	11.67 (9.54, 17.29)	14.30 (10.58, 17.77)	1.21	
Min - Max	1.67 - 32.29	2.50 - 32.39		
**SDMA**				0.80

6 – 17 years	**Control** 6 – 17 years **(N=57)**	**ASD**-6 – 17 years **(N=126)**	**ASD/Control ratio**	**p value**
Mean (SD)	30.93 (13.53)	30.89 (13.53)	1.00	
Median (Q1, Q3)	30.92 (22.37, 37.82)	29.08 (20.44, 37.31)	0.97	
Min - Max	7.07 - 68.91	4.09 - 70.26		
**TMAO**				0.96
Mean (SD)	3.24 (2.44)	3.21 (2.46)	1.00	
Median (Q1, Q3)	2.58 (1.48, 4.53)	2.84 (1.40, 4.85)	1.10	
Min - Max	0.00 - 9.30	0.01 - 10.19		
**IS**				0.39
Mean (SD)	68.85 (39.42)	63.75 (40.38)	0.93	
Median (Q1, Q3)	58.27 (40.36, 100.61)	60.48 (28.22, 85.99)	1.04	
Min - Max	3.57 - 168.60	3.16 - 193.60		
**PCS**				0.69
Mean (SD)	51.39 (44.33)	57.23 (47.94)	1.11	
Median (Q1, Q3)	34.67 (19.75, 67.19)	46.65 (15.49, 85.86)	1.35	
Min - Max	1.27 - 195.67	0.13 - 188.20		

Similar data for older age group showing consistency with trends in PCS and IS ratios, but with reduced TMAO differences.

Table S3. Uremic toxin concentrations stratified by sex (ASD boys vs. ASD girls).

	**Boys (N=161)**	**Girls (N=71)**	**ASD/Control ratio**	**p value**
**ADMA**				0.82
Mean (SD)	14.99 (6.86)	15.51 (7.26)	1.03	
Median (Q1, Q3)	14.39 (10.67, 18.32)	14.41 (11.03, 20.10)	1.00	
Min - Max	1.67 - 34.36	2.49 - 34.34		
**SDMA**				0.53
Mean (SD)	33.12 (15.52)	35.00 (16.28)	1.06	
Median (Q1, Q3)	30.71 (22.61, 40.86)	31.14 (24.79, 44.81)	1.01	
Min - Max	4.09 - 80.21	7.07 - 79.83		
**TMAO**				0.37
Mean (SD)	3.30 (2.59)	2.93 (2.48)	0.89	
Median (Q1, Q3)	3.01 (1.37, 4.95)	2.58 (0.50, 4.10)	0.86	
Min - Max	0.00 - 10.52	0.01 - 10.38		
**IS**				0.30
Mean (SD)	64.64 (40.37)	69.96 (39.83)	1.08	
Median (Q1, Q3)	53.59 (30.34, 92.06)	66.50 (39.59, 100.61)	1.24	
Min - Max	3.16 - 172.30	3.57 - 193.60		
**PCS**				0.20
Mean (SD)	52.17 (43.49)	63.01 (51.76)	1.21	

	Boys (N=161)	Girls (N=71)	ASD/Control ratio	p value
Median (Q1, Q3)	40.75 (17.12, 79.21)	46.65 (20.02, 101.98)	1.14	
Min - Max	0.13 - 174.60	1.48 - 195.67		

Girls with ASD exhibit higher PCS concentrations and total burden compared to boys, with altered IS/PCS ratios.

Table S4. Uremic toxin concentrations stratified by CARS severity (CARS <36 and >36.5).

	Control (N=71)	CARS<36 (N=58)	CARS>36.5 (N=34)	p value		
	0	1	2	0:1	0:2	1:2
**ADMA**				0.26	0.66	0.65
Mean (SD)	14.23 (7.03)	15.74 (6.77)	14.74 (7.62)			
Median (Q1, Q3)	12.70 (9.73, 18.74)	15.14 (11.28, 18.31)	14.83 (9.21, 19.79)			
Min - Max	1.67 - 33.16	4.53 - 34.34	2.50 - 34.36			
**SDMA**				0.99	0.53	0.42
Mean (SD)	32.25 (13.83)	32.47 (12.82)	31.72 (18.24)			
Median (Q1, Q3)	31.32 (23.31, 38.98)	30.03 (25.00, 38.91)	28.53 (17.61, 44.83)			
Min - Max	7.07 - 68.91	11.09 - 64.63	4.09 - 71.67			
**TMAO**				0.57	0.30	0.62
Mean (SD)	3.52 (2.72)	3.27 (2.64)	2.90 (2.44)			
Median (Q1, Q3)	3.09 (1.48, 5.19)	2.50 (1.56, 4.99)	2.60 (0.48, 5.01)			
Min - Max	0.00 - 10.38	0.01 - 10.19	0.01 - 9.75			
**IS**				0.35	0.16	0.59
Mean (SD)	71.04 (40.71)	63.28 (37.92)	61.22 (44.52)			
Median (Q1, Q3)	63.80 (39.77, 103.18)	60.74 (27.44, 86.11)	52.58 (26.30, 88.25)			
Min - Max	3.57 - 168.60	9.12 - 172.30	3.16 - 193.60			
**PCS**				0.96	0.58	0.50
Mean (SD)	52.82 (43.65)	54.64 (47.22)	62.54 (51.24)			
Median (Q1, Q3)	37.74 (20.02, 80.02)	49.66 (13.34, 79.29)	53.37 (16.70, 94.26)			

PCS levels rise with severity, IS and TMAO decline. Ratio patterns align with metabolic shift hypotheses.
